# *Ex-vivo* training model for laparoendoscopic single-site surgery

**DOI:** 10.4103/0972-9941.72398

**Published:** 2011

**Authors:** Sashi S Kommu

**Affiliations:** Department of Urology, University Hospital North Staffordshire, Stoke-on-Trent, U.K.

**Keywords:** Box, laparoendoscopic, laparoscopic, learning curve, laparoendoscopic single-site surgery, natural orifice translumenal endoscopic surgery, port, programmes, R-port, single, single-site, surgery, training, urology

## Abstract

**BACKGROUND:**

Laparoendoscopic single-site surgery (LESS) has recently been applied successfully in the performance of a host of surgical procedures. Preliminary consensus from the experts is that this mode of surgery is technically challenging and requires expertise. The transition from trainee to practicing surgeon, especially in complex procedures with challenging learning curves, takes time and mentor-guided nurturing. However, the trainee needs to use platforms of training to gain the skills that are deemed necessary for undertaking the live human case.

**OBJECTIVE:**

This article aims to demonstrate a step-by-step means of how to acquire the necessary instrumentation and build a training model for practicing steeplechase exercises in LESS for urological surgeons and trainees. The tool built as a result of this could set the platform for performance of basic and advanced skills uptake using conventional, bent and articulated instruments. A preliminary construct validity of the platform was conducted.

**MATERIALS AND METHODS:**

A box model was fitted with an R-Port^™^ and camera. Articulated and conventional instruments were used to demonstrate basic exercises (e.g. glove pattern cutting, loop stacking and suturing) and advanced exercises (e.g. pyeloplasty). The validation included medical students (M), final year laparoscopic fellows (F) and experienced consultant laparoscopic surgeons (C) with at least 50 LESS cases experience in total, were tested on eight basic skill tasks (S) including manipulation of the flexible cystoscope (S1), hand eye coordination (S2), cutting with flexible scissors (S3), grasping with flexible needle holders (S4), two-handed maneuvers (S5), object translocation (S6), cross hand suturing with flexible instruments (S7) and conduction of an *ex-vivo* pyeloplasty.

**RESULTS:**

The successful application of the box model was demonstrated by trainee based exercises. The cost of the kit with circulated materials was less than £150 (Pounds Sterling). The noncamera handling skills (S2–S8) of the *ex-vivo* training model for LESS can distinguish between laparoscopically naïve fellows and experienced consultants in LESS. S4–S8 showed the highest level of construct validity, by accurately differentiating among the M, F and C groups.

**CONCLUSION:**

LESS requires a significant amount of skill and has an inherent steep learning curve. The *ex-vivo* model described provides a cost-effective means that a trainee or training unit can build for optimising preliminary skill acquisition in LESS for urological trainees. It has construct validity in several tasks. Such platform models should be tested further with an emphasis on rapid sequence uptake of optimal skills, prior to undertaking the live human case.

## INTRODUCTION

The advantages of laparoscopic surgery in comparison to open surgery are well established.[1,2] This approach led to decreased pain, morbidity, hospital duration and cost of many commonly performed procedures as compared to the “open” surgical counterpart. Following the first laparoscopic nephrectomy by Clayman *et al*. in 1991, minimally invasive urological surgery has gained significant momentum.[3] Currently, laparoscopy has assumed a central role in the surgical treatment of renal, adrenal, prostatic, bladder and some cases of testicular diseases. Short-term measures of convalescence continue to be favourable, while oncologic outcomes are comparable to contemporary cohorts of patient treated with open surgical treatment.[4]

Whereas laparoscopy is well recognised in decreasing surgical morbidity, it still requires three to five spaced incisions each at least 1–2 cm in length. Triangulation of instrumentation is one of the fundamental concepts of conventional laparoscopic surgery. Placement of trocars in a relatively precise fashion is needed to allow intracorporeal spacing of the instruments to permit tissue dissection, traction, extirpation and/or reconstruction. Triangulation also reduces the frequency of “collision” of laparoscopic instruments extra and intracorporeally. With conventional laparoscopy, it is well recognised that each working port carries additional risks of morbidity such as bleeding, hernia and/or internal organ damage, and incrementally reduces favourable cosmetic outcomes.[5,6] Cosmesis is naturally important in procedures involving paediatric patients, and in many cases, an expectation of adult patients.[7] The quest to make minimally invasive techniques even more “minimal” has generated a drive within the surgical community to explore novel ways of achieving this. This led to surgeons attempting to either decrease the number of trocars placed through the abdominal wall or eliminate them completely leading to the birth of several approaches, e.g. natural orifice translumenal endoscopic surgery (NOTES), single-incision laparoscopic surgery (SILS), single port access surgery and one port umbilical surgery (OPUS) or E-NOTES. The most recent consensus on nomenclature involves the term laparoendoscopic single-site surgery (LESS).

With respect to skill acquisition, preliminary consensus from the experts is that this mode of surgery is technically challenging and requires expertise. The transition from trainee to practicing surgeon, especially in complex procedures with challenging learning curves, takes time and mentor-guided nurturing. However, the trainee needs to use platforms of training to gain the skills that are deemed necessary for undertaking the live human case. This article aims to act as a guide towards building a training model for practicing steeplechase exercises in LESS for urological surgeons and trainees. Such a platform could act as a useful supplementary tool for basic skill acquisition for performance of basic and advanced skills uptake using conventional, bent and articulated instruments.

## MATERIALS AND METHODS

**Figure 1:**
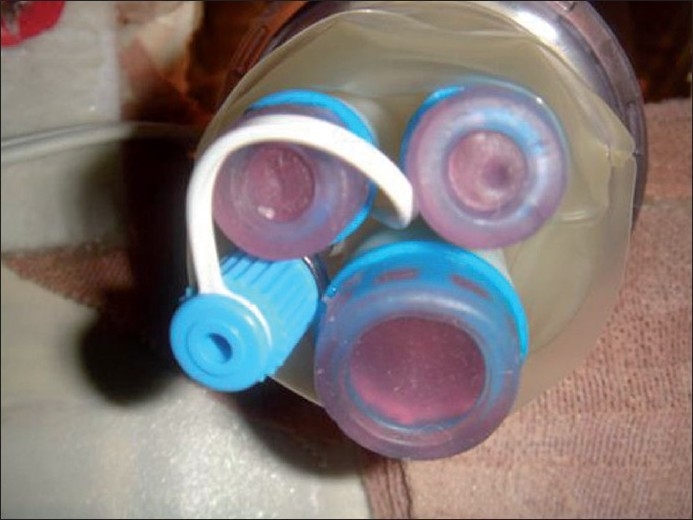
The R-Port™

The following is a stepwise systematic approach to build a robust platform for skill acquisition in LESS. Raw materials required include flexible instruments, longitudinal cardboard box, a Gel Port™, i.e. the one used in hand-assisted laparoscopic surgery, a multichannel port (e.g. R-Port™), flexible cystoscope or other camera device, rolled cloth × 2 and masking tape. The R-Port is a multi-instrument access port. It has three channels to permit a maximum of three instruments to be used simultaneously [Figure 1]. Two of the channels involve 5-mm gel valves and one has a 12-mm gel valve. There is a separate portal for insufflation of gas. The tear-resistant thermoplastic elastomer valves allow individual inserted instruments to be operated using an independent fulcrum.

**Figure 2:**
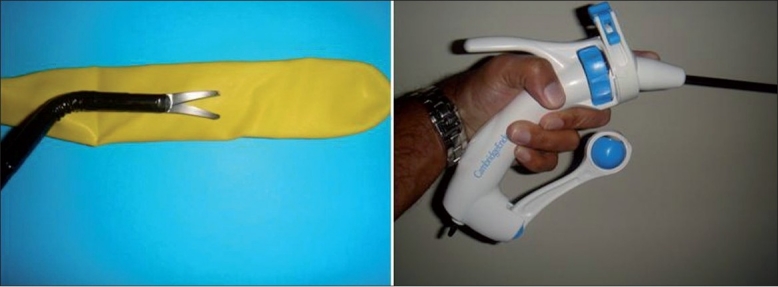
Flexible instruments

Currently, commercially available instruments can be broadly divided into articulating laparoscopic graspers and shears (Real Hand™, Novare Surgical Systems, Cupertino, CA, USA and Autonomy Laparo-angle™, Cambridge Endo, Framingham, MA, USA), endoshears (Cambridge Endo), and laparoscopic needle drivers (Cambridge Endo). Autonomy™ Laparo-Angle™ Instruments from Cambridge Endo represent another means of flexible instrumentation [Figure 2]. The articulating tips offer seven degrees of freedom of motion. These instruments map, in exact proportion, the motion of the hand holding the instrument. There is an axial rotation knob and tip orientation locking mechanism. These mechanisms can be controlled with a solitary hand rendering precise control of the instrument tip. The movement of the tip is in tandem with movement of the surgeon’s palm. When turning the axial rotation knob in handle, the tip turns 360° around its axis at any angle. The instruments are 5 mm in diameter.

**Figure 3:**
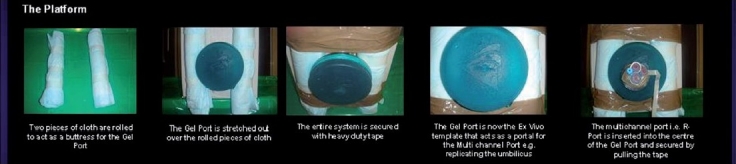
The Building of the box kit platform

Having acquired the necessary raw materials, the next step is to build the platform to practice the steeplechase exercises. A longitudinal box is cut at the top such that the trainee can look directly into it to allow for direct three-dimensional view of the target task. The use of articulated instruments as dictated by LESS requires familiarisation, which is best done by direct visualisation prior to viewing on the monitor with its restricted two-dimensional view. Two pieces of cloth are rolled to act as a buttress for the Gel Port [Figure 3]. The Gel Port is stretched out over the rolled pieces of cloth and the entire system is secured with heavy-duty tape. The Gel Port will then be an *ex vivo* template that acts a portal for a further multichannel port and could be seen as replicating an umbilicus at its centre. The multichannel R-Port is then inserted into the centre of the Gel Port and secured. The platform for skill acquisition is now complete.

**Figure 4:**
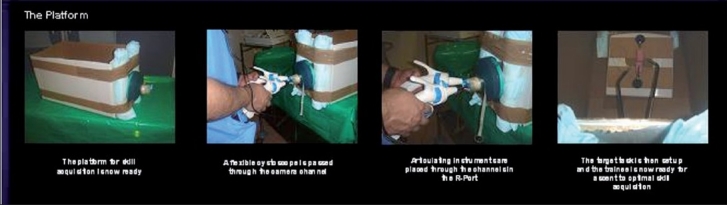
The use of the flexible instruments via the channel. Note that the skill acquisition in terms of use of the instruments does not require a camera in the initial phase when one is trying to get used to the instruments

**Figure 5:**
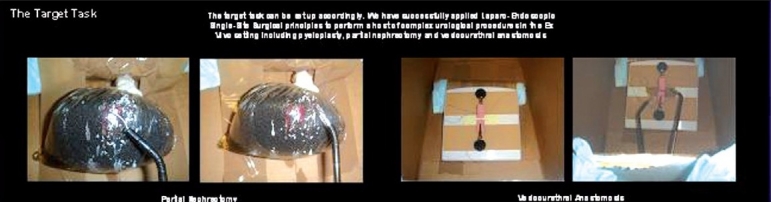
An example of an advanced platform for skill acquisition using the partial nephrectomy model and vesicourethral anastomosis

The next step is to introduce the instruments and to set the target tasks. Articulating instruments are placed into the appropriate channels. Camera(s) can be set up just above the box to permit viewing via a monitor. A flexible cystoscope can be placed via the camera port and linked to a monitor. It is recommended that the trainee first use direct vision over the box to allow for instrument familiarisation prior to undertaking the more challenging two-dimensional view via a camera [Figure 4]. The target task is set up and the trainee can progress under mentor guidance. Following basic skill acquisition such as loop stacking and pattern cutting, the trainee can practice suturing and more advanced tasks [Figure 5]. Here, the target task involves a partial nephrectomy and vesicourethral anastomosis using the continuum-based approach.

We tested this preliminary platform for *ex-vivo* training for skill acquisition in performing a LESS pyeloplasty. A box model was fitted with a multichannel single port and flexible cystoscope. The target task of pyeloplasty involved a level-3 model using a continuum-based approach.[8] Level-1 model was a basic model using a small balloon with an elongated mouth; level-2 consisted of rolled supermarket chicken skin fashioned into a tapered cylindrical structure. Level-3 is the same as level-2 but with added medium for absorption of projection optics and transposition of a template slide of the retroperitoneum. Articulated and conventional instruments were used to perform the necessary pyeloplasty. The main challenge was stated to be instrument collision and narrow window of triangulation using the articulating instruments.[9]

Medical students (M), final year laparoscopic fellows (F), and experienced consultant laparoscopic surgeons (C) with at least 50 LESS cases experience in total, were tested on eight basic skill tasks (S) including manipulation of the flexible cystoscope (S1), hand eye coordination (S2), cutting with flexible scissors (S3), grasping with flexible needle holders (S4), two-handed maneuvers (S5), object translocation (S6), cross hand suturing with flexible instruments (S7) and conduction of a level-3 model pyeloplasty.

## RESULTS

The kit was successfully constructed and tested. Total cost of raw materials required include the cost for flexible instruments, longitudinal cardboard box, a Gel Port, the multichannel port, flexible cystoscope, rolled cloth × 2 and masking tape, which was approximately £1450 (Pounds Sterling). The deployment of donated raw materials reduces the cost of the platform to £120 (Pounds Sterling). Once set up, the kit could be used repeatedly to perform the target tasks. This is considerably cheaper compared to virtual reality platforms that have been quoted as high as £4000 (Pounds Sterling).

As LESS is a relatively novel approach with few centres of excellence practicing the techniques, the total number of participants was relatively small, i.e. (*n* = 6). It included medical students (M) (*n* = 2), final year laparoscopic fellows (F) (*n* = 2), and experienced consultant laparoscopic surgeons (C) (*n* = 2). All the fellows assisted in at least five cases each. None of the medical students had prior LESS or any form of laparoscopic experience. There was a small but insignificant difference in scores between the three groups in terms of cross hand manipulation of the flexible cystoscope (S1). The F and C groups had similar scores in hand eye coordination (S2), cutting with flexible scissors (S3 grasping with flexible needle holders (S4). Their scores were significantly higher than the M group (*P*=0.001 for S2 and S3; *P*=0,01 for S4). There was considerable difference in scores between the three groups in the tasks of two-handed maneuvers (S5), object translocation (S6), cross hand suturing with flexible instruments (S7) and conduction of a level-3 model pyeloplasty (S4). The difference in scores between F and C was greater than a factor of 2 (*P* = 0.001). The noncamera handling skills (S2–S8) of the *ex-vivo* training model for LESS can distinguish between laparoscopically naïve fellows and experienced consultants in LESS. S4–S8 showed the highest level of construct validity, by accurately differentiating among the M, F and C groups.

## DISCUSSION

Trainees interested in minimally invasive surgery, and indeed their mentors, are under increasing pressure to acquire, maintain and transfer optimal laparoscopic and robotic urological skills. In an era where technically challenging approaches such as LESS are being undertaken in several centres of excellence, there is increasing demand for rapid sequence acquisition of basic laparoscopic skills from the outset. With respect to skill acquisition, preliminary consensus from the experts is that the LESS approach is technically more challenging and requires more expertise as compared to conventional laparoscopy.[10] The triangulation required for safe and successful completion of a given task via a single multichannel port poses ergonomic and technical challenges. There is a requirement for optimal skill sets with a narrow margin for error as compared to conventional laparoscopic surgery. One way of tackling this is to optimise skill acquisition prior to undertaking the live human case. Surgical simulators could provide opportunities for target task practice by repetition. Despite evidence that mastering skills on a simulator minimises operating room time in some cases,[11] there are limitations to simulators in that they do not always replicate the task platform and they are expensive, making practice at home difficult. Proponents of simulators argue that although *ex-vivo* inanimate training platforms result in significant acquisition of laparoscopic skills, there is difficulty in performing objective quantification of skills, thus forcing human supervision and assessment.[12] Mentor-driven target tasking and skill acquisition in *ex-vivo* platforms are feasible. The LESS platform in this study represents a cost-effective platform that could be made in the home setting. The kit has construct validity for more complex tasks including *ex-vivo* pyeloplasty. The utility of the training modules by novices from the outset is on the upsurge recently with the realisation that skill acquisition can be had in a rapid, safe and cost-effective manner.[13]

## CONCLUSION

The *ex-vivo* model described provides a cost-effective means upon which preliminary skill acquisition could be acquired and used by trainees for skill acquisition in training programmes.
